# Oral health-related quality of life and loneliness: results based on a cross-sectional survey

**DOI:** 10.1186/s13690-024-01347-9

**Published:** 2024-07-29

**Authors:** Ammar Abdullah, Hans-Helmut König, André Hajek

**Affiliations:** Department of Health Economics and Health Services Research, University Medical Center Hamburg-Eppendorf, Hamburg Center for Health Economics, Hamburg, Germany

**Keywords:** Oral health, Oral health-related quality of life, Loneliness, Social exclusion, Social isolation

## Abstract

**Background:**

The aim of this study was to clarify the link between oral health-related quality of life (independent variable) and loneliness (outcome) among the general adult population (also stratified by sex).

**Methods:**

Data were taken from a quota-based survey of the German general adult population (representative in terms of state, sex and age group), with *n* = 5,000 individuals (mean age was 46.9 years, SD: 15.3 years, ranging from 18 to 74 years). Oral health-related quality of life was quantified using the OHIP-G5. Loneliness was quantified using the De Jong Gierveld tool. Multiple linear regressions were conducted.

**Results:**

After adjusting for several covariates, multiple linear regressions revealed that poor oral health-related quality of life is associated with higher loneliness levels in the total sample (β = 0.12, *p* < 0.001). Such associations were also found in both sexes (men: β = 0.12, *p* < 0.001, women: β = 0.12, *p* < 0.001).

**Conclusion:**

Study findings showed an association between oral health-related quality of life and loneliness. Such knowledge is important for addressing individuals at risk for higher loneliness levels. Future research is required to clarify the underlying mechanisms.

**Supplementary Information:**

The online version contains supplementary material available at 10.1186/s13690-024-01347-9.

**Table:** 

**Text box 1. Contributions to the literature**
• There is a limited number of studies examining the link of oral health-related quality of life and loneliness (also stratified by sex).
• Our aim was therefore to address this gap in knowledge.
• We found an association between oral health-related quality of life and loneliness (total sample and in both sexes).
• Such knowledge is important for addressing individuals at risk for higher loneliness levels.

## Background

Loneliness is the emotional and psychological experience of being isolated and disconnected from social interactions due to an unfulfilled desire for relationships [1]. It includes the feeling of being isolated, misunderstood or feeling distant from others, even when there is the possibility of physical interaction. Loneliness affects, among other things, health [2], quality of life [3] and happiness [4], leading to negative consequences in various areas of life. It is also important to note that loneliness can affect work productivity [5].

Loneliness is a huge challenge since it is associated with later morbidity and mortality [6]. Numerous factors that contribute to loneliness among older adults have been investigated. For instance, spousal loss is clearly linked to higher levels of loneliness [7]. Other determinants of loneliness have also been extensively studied [8].

Thus far, there is limited knowledge regarding the association between oral health-related quality of life and loneliness (e.g., [9]). For example, a former study focused on the association between oral health-related quality of life and loneliness [9]. To this end, they used data from the representative English Longitudinal Study of Ageing (covering respondents aged 50 years and above). They found that poor oral-health related quality of life is associated with higher loneliness levels among older adults. Another study [10] found that oral health status was negatively correlated with loneliness among the migrant elderly following children (i.e., older adults relocating to urban areas alongside their children in order to attend to the needs of their grandchildren) in China (Weifang). In a further previous study [11], the focus was on investigating the relationship between oral health-related quality of life and feelings of loneliness. The study utilized data obtained from a nationally representative survey involving a sample size of 3,075 individuals living in Germany. The findings revealed an association between lower oral health-related quality of life and increased feelings of loneliness.

In many previous studies, sex-specific analyses are neglected [12]. As a result, possible differences or potential influences that sex may have on certain variables or outcomes may not be adequately considered. This could lead to important details or sex-specific patterns in the data being overlooked. Therefore, it is important to integrate sex analysis into research to gain a more comprehensive understanding of the specific effects or differences between sexes in relation to the variables under consideration. This is crucial in order to gain informed insights and draw appropriate conclusions or recommendations that better take into account the diversity of the populations studied.

Due to the restricted knowledge (particularly related to sex-stratified analyses), we aimed to address this gap in knowledge. More explicitly, our first aim is to shed light on the association between oral health-related quality of life and loneliness among the general adult German population.

Our second aim was to examine oral health-related quality of life and loneliness stratified by sex. We also stratify our regressions by sex for the following reasons: We assume that, unlike women, men with poor oral health-related quality of life may not experience such a deep sense of impairment or shame [13]. The feeling of shame could cause a person to withdraw more, and this withdrawal can in turn lead to the person feeling lonely. Previous research showed that women are more influenced by societal ideals of beauty and media images [14], which may lead them to perceive poor oral health-related quality of life as particularly distressing. This potential pressure may affect women’s general self-esteem which could eventually increase their loneliness. Consequently, loneliness may be more pronounced in women than in men when they are report a poor oral health-related quality of life. On the other side, one may assume that the relationship between oral health-related quality of life and loneliness is particularly strong among men. A potential explanation may be that women have stronger social networks compared to men [15]. Men tend to talk less about their problems (such as poor oral health-related quality of life) or seek support when in need [16]. This may lead to a greater withdrawal from social contacts among men.

Although there are possible explanations for the fact that the association between oral health-related quality of life and loneliness could be stronger for both women and men, overall we assume that the aforementioned association is stronger for women. The main reason for this could be that the ideals of beauty are more pronounced in women. In the face of a bad poor oral health-related quality of life, this social pressure could lead to greater loneliness in women.

With regard to rationale of this study, knowledge about an association between oral health-related quality of life and loneliness is important because oral health-related quality of life is modifiable (by individuals and dental practitioners) [12], it ultimately points to ways to combat loneliness.

## Methods

### Sample

The data was collected through a comprehensive online survey of a representative sample of 5,000 people living in Germany and aged between 18 and 74. The respondents completed the questionnaire themselves online. If they had any questions about the content, they could contact us by e-mail. In the event of technical difficulties, they could contact the market research company.

This extensive data collection took place between August and September 2023, with the reputable market research company Bilendi, certified to ISO standard 26,362, taking responsibility for selecting participants from a carefully selected online pool. The selection criteria were meticulously defined to use specific quotas to ensure that the sample accurately reflects the age range, sex distribution and geographical spread of the German adult population aged 18 to 74. This careful methodology ensured the closest possible representation of these demographic characteristics.

Prior to participation, all participants gave their informed consent. In addition, the study received ethical approval from the local ethics committee for psychology of the University Medical Center Hamburg-Eppendorf (LPEK-0629).

### Dependent variable: Loneliness

A 6-item version of the De Jong Gierveld short scale [17] for loneliness was used to quantify loneliness. The scale ranges from 0 to 6, with higher scores indicating a greater degree of loneliness. This numerical scale serves as a measurement tool to assess and quantify a person’s level of loneliness. It allows a clear assessment of the intensity or severity of loneliness based on the assigned values. The scale thus provides a structured method to capture and understand the extent of loneliness, enabling a more precise assessment of a person’s feelings of loneliness.

Respondents were asked to rate six statements about their personal situation, using a three-point scale: “Yes”, “More or less” and “No”. These statements included sentences such as “I miss contact with people with whom I feel comfortable” and “There are enough people with whom I feel a close connection”. The participants were asked to use these statements to assess their subjective perception and evaluation of their social ties and relationships. These specific statements aimed to gain insight into the feeling of connectedness and closeness to other people, as well as the extent of the respondents’ social well-being. Cronbach’s alpha was 0.80 in our present study.

### Independent variables of interest: Oral health-related quality of life

In this research, the primary independent variable assessed was oral health-related quality of life. The widely used Oral Health Impact Profile (OHIP-G5) was used for this purpose [18]. This instrument consists of five original scales that assess different aspects of oral health-related quality of life: functional limitations, physical impairments, physical pain, psychological discomfort and social impairments. The OHIP-G5 is divided into four different dimensions: (i) oral function, (ii) orofacial pain, (iii) appearance and (iv) psychosocial impact. The OHIP-G5 has been shown to have positive psychometric properties [19]. This scale ranges from 0 to 20, with higher scores indicating a lower quality of life in relation to oral health. The internal consistency of the OHIP-G5 in our current study was good, with a Cronbach’s alpha coefficient of 0.85. This instrument allowed a comprehensive assessment of different aspects of oral health and its impact on respondents’ quality of life, with a solid internal consistency of the data collected.

### Covariates

The choice of the covariates was guided by theoretical considerations and prior research [8]. More precisely, it was adjusted for several sociodemographic and health-related covariates in regression analysis. It was adjusted for the following several sociodemographic factors in regression analysis: age (in years), sex (male; female; diverse), state (Baden-Württemberg; Bavaria; Berlin; Brandenburg; Bremen; Hamburg; Hesse; Mecklenburg-Western Pomerania; Lower Saxony; North Rhine-Westphalia; Rhineland-Palatinate; Saarland; Saxony; Saxony-Anhalt; Schleswig-Holstein; Thuringia), marital status (single; divorced; widowed; living separated: married or in partnership; living together: married or in partnership), education (low education; middle education; high education) and employment status (full-time employment; retired; other professional activity) according to the Comparative Analysis of Social Mobility in Industrial Nations (CASMIN) [20].

It was also adjusted for these two health-related factors: Self-rated health (single item measure from 1 (very poor) to 5 (very good)) and the number of chronic diseases (in each case: present of a chronic condition = 1; absence = 0; sleep disorder; thyroid disease; diabetes; asthma; heart disease (including heart failure, cardiac insufficiency); cancer; stroke; migraine; high blood pressure; depressive illness; dementia; joint disease (including osteoarthritis, rheumatism); chronic back pain; burnout; other illness).

### Statistical analysis

Sample characteristics are first shown. Effect sizes (in terms of Pearson’s r) were computed for the link between oral health-related quality of life and loneliness (also stratified by sex). Pearson’s r can be interpreted as follows [21]: 0.10 to 0.29 (small correlation), 0.30 to 0.49 (medium correlation) and 0.50 or higher (large correlation).

To investigate the association between oral health-related quality of life (independent variable) and loneliness (outcome), multiple linear regressions were conducted (also stratified by sex). The effect sizes (in terms of partial eta² values) were also reported. They can be interpreted as follows [21]: 0.01 as “small”, 0.06 as “medium, and 0.14 as “large”.

We also conducted a robustness check where we created oral health-related quality of life quartiles (0 if OHIP-G5 score equaled 0; 1 if OHIP-G5 score equaled 1; 2 if OHIP-G5 score ranged from 2 to 4; 3 if OHIP-G5 score was 5 or higher) to check whether the association between oral health-related quality of life and loneliness is roughly linear.

Statistical significance was defined as *p* value of 0.05 or smaller. Stata 17.0 (Stata Corp., College Station, Texas) was used to conduct statistical analyses.

## Results

### Sample characteristics

Sample characteristics for the total analytical sample are shown in Table 1. In the total analytical sample, average age equaled 46.9 years (SD: 15.3 years). Moreover, 50.8% of the individuals were female. Average loneliness score was 3.1 (SD: 2.1). Furthermore, average oral health-related quality of life was 2.7 (SD: 3.9). Further details are provided in Table 1.

**Table 1:** Sample characteristics

**Sex**	Mean (SD) / *n* (%)
Male	2451 (49.0%)
Female	2540 (50.8%)
Diverse	9 (0.2%)
**Age**	46.9 (15.3)
**State**	
Baden-Württemberg	649 (13.0%)
Bavaria	804 (16.1%)
Berlin	196 (3.9%)
Brandenburg	152 (3.0%)
Bremen	50 (1.0%)
Hamburg	101 (2.0%)
Hesse	406 (8.1%)
Mecklenburg-Western Pomerania	99 (2.0%)
Lower Saxony	492 (9.8%)
North Rhine-Westphalia	1067 (21.3%)
Rhineland-Palatinate	254 (5.1%)
Saarland	49 (1.0%)
Saxony	248 (5.0%)
Saxony-Anhalt	152 (3.0%)
Schleswig-Holstein	142 (2.8%)
Thuringia	139 (2.8%)
**Marital status**	
single/divorced/widowed/living separated: married or in partnership	2107 (42.1%)
Living together: Married or in partnership	2893 (57.9%)
**Education**	
Low education	533 (10.7%)
Middle education	2987 (59.7%)
High education	1480 (29.6%)
**Employment status**	
Full-time employed	2418 (48.4%)
Retired	1000 (20.0%)
Others	1582 (31.6%)
**Self-rated health** (1 = very bad to 5 = very good)	3.6 (0.8)
**Count score (from 15 chronic diseases)**	1.7 (1.8)
**Oral health-related quality of life**	2.7 (3.9)
**Difficulty chewing**	0.6 (1.0)
**Less flavor in food**	0.5 (0.9)
**Painful aching**	0.6 (0.9)
**Uncomfortable about appearance**	0.7 (1.1)
**Difficulty doing your usual job**	0.3 (0.8)
**Loneliness**	3.1 (2.1)

We also examined the effect size (in terms of Pearson’s r) between oral health related quality of life and loneliness among the total sample. Pearson’s r between those variables was 0.29 (*p* < 0.001) among men. Moreover, it was *r* = 0.27 (*p* < 0.001) among women and *r* = 0.07 (*p* = 0.87) among diverse individuals.

### Regression analysis

Findings of regressions are displayed in Table 2 (total sample and stratified by sex). R² value was 0.20 (total sample). Stratified by sex, R² was 0.23 in men and 0.18 in women. There was an association between poor oral health-related quality of life and higher loneliness levels among the total sample (β = 0.12, *p* < 0.001, 95% CI: 0.11 to 0.13) and among both sexes (men: β = 0.12, *p* < 0.001, 95% CI: 0.11 to 0.14; women: β = 0.12, *p* < 0.001, 95% CI: 0.10 to 0.14). The interaction term (sex x oral health-related quality of life) did not achieve statistical significance (β = 0.01, *p* = 0.36, 95% CI: -0.01 to 0.04). Partial eta² values of oral health-related quality of life was 0.05 (among the total sample), 0.06 (among men), and 0.04 (among women), mainly reflecting medium effect sizes. Furthermore, multiple linear regressions using the four dimensions (rather than OHIP G5) as key independent variables are shown in the Supplementary Tables 1 to 4, with the greatest effect sizes of the four dimensions (i.e., mainly small to medium effect sizes in terms of partial eta² values) for the dimension psychosocial impact.

**Table 2:** Determinants of loneliness (total sample and stratified by sex). Results of multiple linear regressions

	(1)	(2)	(3)
Independent variables	Loneliness - Total sample	Loneliness - Men	Loneliness - Women
Oral health-related quality of life	0.12***	0.12***	0.12***
	(0.01) [0.11 to 0.13]	(0.01) [0.11 to 0.14]	(0.01) [0.10 to 0.14]
Covariates	✓	✓	✓
Observations	5000	2451	2540
R²	0.20	0.23	0.18

Unstandardized beta-coefficients are reported, robust standard errors in parentheses, 95% CI in square brackets; *** *p* < 0.001, ** *p* < 0.01, * *p* < 0.05, + *p* < 0.10; Covariates include sex (if applicable), age, state, employment status, marital status, education, self-rated health and number of chronic conditions

In a robustness check, oral health-related quality of life quartiles were used (see Table 3). These findings show that higher oral health-related quality of life quartiles (reflecting poorer oral health-related quality of life) were associated with higher loneliness levels (compared to the lowest oral health-related quality of life quartile). Particularly the highest oral health-related quality of life quartile had medium effect sizes (partial eta²-values) reflecting the importance of very poor oral health-related quality of life for loneliness. More details are given in Table 3. All covariates of the regressions presented in Tables 2 and 3 (and the model with the interaction term) are displayed in Supplementary Tables 5, 6 and 7.

**Table 3:** Determinants of loneliness (total sample and stratified by sex). Results of multiple linear regressions (with quartiles for oral health-related quality of life)

	(1)	(2)	(3)
Independent variables	Loneliness - Total sample	Loneliness - Men	Loneliness - Women
Oral health-related quality of life: - Second quartile (Ref.: Lowest quartile)	0.35***	0.25*	0.44***
	(0.09) [0.18 to 0.53]	(0.12) [0.004 to 0.49]	(0.13) [0.19 to 0.69]
- Third quartile	0.68***	0.66***	0.73***
	(0.07) [0.54 to 0.82]	(0.10) [0.46 to 0.85]	(0.10) [0.53 to 0.92]
- Highest quartile	1.27***	1.34***	1.22***
	(0.07) [1.13 to 1.41]	(0.10) [1.14 to 1.53]	(0.11) [1.01 to 1.42]
Covariates	✓	✓	✓
Observations	5000	2451	2540
R²	0.20	0.23	0.19

Unstandardized beta-coefficients are reported, robust standard errors in parentheses; *** *p* < 0.001, ** *p* < 0.01, * *p* < 0.05, + *p* < 0.10; Covariates include sex (if applicable), age, state, employment status, marital status, education, self-rated health and number of chronic conditions

In a further robustness check, household net income category (13 categories from “below 500 Euro” to more than 8,000 Euro) was added to our main model. However, the associations of interest remained nearly the same in terms of significance and effect size: There was still an association between poor oral health-related quality of life and higher loneliness levels among the total sample (β = 0.12, *p* < 0.001, 95% CI: 0.10 to 0.13) and among both sexes (men: β = 0.12, *p* < 0.001, 95% CI: 0.10 to 0.13; women: β = 0.12, *p* < 0.001, 95% CI: 0.10 to 0.14).

## Discussion

### Main findings

Based on a large representative sample, this study aimed to investigate the relationship between oral health-related quality of life (independent variable) and loneliness (outcome) in the general adult population in Germany, also differentiating by sex. Using multiple linear regressions, an association was found between poor oral health-related quality of life and higher levels of loneliness in both the overall sample and in both sexes (differences between both sexes were thus not found), suggesting that people with poor oral health-related quality of life may be at higher risk of loneliness. The effect size was mostly medium in size. The findings suggest that a targeted improvement in oral health may also have a positive impact on social and emotional well-being. The sex differences within these associations were also looked at in more detail to provide nuanced insights into possible sex-specific effects.

### Relation to previous research and potential explanations

In research on oral health-related quality of life and loneliness, there is a diverse landscape of studies from different regions of the world. A majority of these studies are from European countries [22, 23], possibly due to the high sensitivity and interest in health issues in this region. There is also some work from Asia [23, 24] that provides a more global insight into this topic, as well as a single study from South America [25] that brings additional perspectives. These research studies vary in their methodology: they rely primarily on cross-sectional data, meaning that they collect data at one point in time to examine relationships. However, one notable study has used both cross-sectional and longitudinal data [9]. Some studies are based on representative samples of the population [12], allowing a broader perspective on the topic, while others examine more specific groups such as hospital patients [26], allowing more detailed insights into specific population segments.

In terms of the main findings of this research literature, there is an overwhelming trend: The majority of studies identify a clear link between poorer oral health and higher levels of loneliness [22, 24, 26, 27]. In contrast, one study did not find such an association when they performed regressions [22]. Interestingly, another study found that there was an association between these factors over time in both cross-sectional and longitudinal analyses, suggesting a consistent relationship between oral health and loneliness [9]. However, it is important to note that none of the works reviewed explored potential sex differences in relation to this topic in more detail. Consideration of sex differences may be crucial to gain a more comprehensive understanding of the impact of oral health on feelings of loneliness and potentially develop differentiated approaches to prevention and intervention. In line with prior research [12], we found an association between oral health-related quality of life and loneliness. We extend prior research [12] by showing such an association for both women and men.

The topic of why oral health-related quality of life is related to loneliness arises. One possible explanation could be that there is a positive link between oral health-related quality of life and mental well-being [28]. This in turn could have an impact on feelings of loneliness [29], as good mental health is often associated with lower levels of loneliness. It is hypothesized that oral health-related quality of life may play a role in mental health and that better dental and oral health may promote mental well-being and facilitate social interactions, which in turn may reduce feelings of loneliness. Previous studies have shown a link between oral health-related quality of life and the condition of being homebound, which may increase loneliness [23]. Additionally, individuals’ sense of self-worth and satisfaction may be diminished by emotions of shame and stigmatization brought on by poor oral health-related quality of life (which may be viewed by others as a proxy for poor socioeconomic position) [30]. As a result, loneliness may also be reported by individuals [31]. Additional justifications for such a relationship between oral health-related quality of life and loneliness in both sexes include the following: People with poor oral health-related quality of life may withdraw socially as they may feel embarrassed about their appearance or because they experience pain. This withdrawal can lead to feelings of loneliness. Awareness that others in the same age group may have healthier teeth can contribute to a negative self-image and feelings of disadvantage. People with poor oral health-related quality of life may thus feel worse about their general health compared to others in their age group. Such negative health comparisons can result in feelings of isolation [32]. An alternative explanation could be that impaired oral health can have a detrimental effect on general well-being [33]. These effects could then in turn have an impact on feelings of loneliness [34].

### Strengths and limitations

The data for this study came from a quota-based survey of the general adult population in Germany. This current study is also one of the few to have examined the relationship between oral health-related quality of life and loneliness, differentiating between sexes. Both loneliness and oral health-related quality of life were measured using established and validated instruments. Although the OHIP-G5 is, generally, an appropriate tool for quantifying oral health-related quality of life in relation, other tools, such as the Child Oral Health Impact Profile (COHIP) [35] for younger children and adolescents and the Geriatric Oral Health Assessment Index (GOHAI) [36] for older adults, may be more appropriate for specific age groups.

It is important to emphasize that this study takes a cross-sectional approach, which means that it represents a single point in time. This makes it difficult to determine whether poor oral health-related quality of life influences loneliness or vice versa (directionality). It is possible that people with poor oral health feel lonelier or that loneliness causes people to take less care of their oral health. This direction of causality can be better clarified by using longitudinal data collected over a longer period of time. This could also help to identify appropriate interventions or preventive measures to improve both oral health and feelings of connectedness.

## Conclusion and future research

Study findings showed an association between oral health-related quality of life and loneliness (total sample and both sexes). This is important to address individuals at risk for high loneliness. This is important because loneliness can increase stress and psychological distress, which in turn can have a negative impact on overall health. Future research should be done on the moderating (such as education) and mediating factors (such as general self-esteem) in the relationship between oral health-related quality of life and loneliness. Moreover, to clarify the directionality between oral health-related quality of life and loneliness, longitudinal studies are required. Furthermore, studies examining the association between oral health-related quality of life and loneliness from other continents is required. The relevance of the relationship between oral health-related quality of life may vary across cultures and continents, as cultural norms, expectations and social practices differ. In some cultures, a poor oral health-related quality of life may be seen as less stigma-related. Therefore, the association between poor oral health, shame and loneliness may be less pronounced in such cultures. Future studies could also explore whether other factors (e.g., age group) play a role in the association between oral health-related quality of life and loneliness.

### Electronic supplementary material

Below is the link to the electronic supplementary material.

Supplementary Material 1

## Data Availability

The datasets generated and/or analysed during the current study are not publicly available due to ethical restrictions but are available from the corresponding author on reasonable request.

## Abbreviations

OHIP: Oral health impact profile
LPEK: Local psychological ethics committee
CASMIN: Comparative Analysis of Social Mobility in Industrial Nations
