# Supplementary material for: Oral health-related quality of life and loneliness: results based on a cross-sectional survey
Source: Arch Public Health. 2024 Jul 29;82:114. doi: 10.1186/s13690-024-01347-9 (PMC11285386; doi:10.1186/s13690-024-01347-9)
Supplement: Supplementary file 1 — Supplementary Material 1 [file 13690_2024_1347_MOESM1_ESM.docx]

Supplementary Table 1. Determinants of loneliness (total sample and stratified by sex). Results of multiple linear regressions.

|  | (1) | (2) | (3) |
| --- | --- | --- | --- |
| Independent variables | Loneliness – Total sample | Loneliness - Men | Loneliness - Women |
|  |  |  |  |
| Dimension oral function: Difficulty chewing | 0.22*** | 0.22*** | 0.22*** |
|  | (0.03)  [0.15 to 0.29] | (0.05)  [0.13 to 0.31] | (0.05)  [0.13 to 0.31] |
| Dimension oral function: less flavor in food | 0.26***  (0.04)  [0.19 to 0.33] | 0.30**  (0.05)  [0.20 to 0.40] | 0.23***  (0.05)  [0.13 to 0.33] |
|  |  |  |  |
| Covariates | ✓ | ✓ | ✓ |
|  |  |  |  |
| Observations | 5000 | 2451 | 2540 |
| R² | 0.19 | 0.22 | 0.17 |

Unstandardized beta-coefficients are reported, robust standard errors in parentheses, 95% CI in square brackets; *** p<0.001, ** p<0.01, * p<0.05, + p<0.10; Covariates include sex (if applicable), age, state, employment status, marital status, education, self-rated health and number of chronic conditions.

Supplementary Table 2. Determinants of loneliness (total sample and stratified by sex). Results of multiple linear regressions.

|  | (1) | (2) | (3) |
| --- | --- | --- | --- |
| Independent variables | Loneliness – Total sample | Loneliness - Men | Loneliness - Women |
|  |  |  |  |
| Dimension orofacial pain: painful aching | 0.39*** | 0.42*** | 0.36*** |
|  | (0.03)  [0.33 to 0.44] | (0.04)  [0.34 to 0.49] | (0.04)  [0.28 to 0.44] |
|  |  |  |  |
| Covariates | ✓ | ✓ | ✓ |
|  |  |  |  |
| Observations | 5000 | 2451 | 2540 |
| R² | 0.18 | 0.21 | 0.16 |

Unstandardized beta-coefficients are reported, robust standard errors in parentheses, 95% CI in square brackets; *** p<0.001, ** p<0.01, * p<0.05, + p<0.10; Covariates include sex (if applicable), age, state, employment status, marital status, education, self-rated health and number of chronic conditions.

Supplementary Table 3. Determinants of loneliness (total sample and stratified by sex). Results of multiple linear regressions.

|  | (1) | (2) | (3) |
| --- | --- | --- | --- |
| Independent variables | Loneliness – Total sample | Loneliness - Men | Loneliness - Women |
|  |  |  |  |
| Dimension appearance: uncomfortable about appearance | 0.36*** | 0.38*** | 0.34*** |
|  | (0.02)  [0.31 to 0.41] | (0.04)  [0.31 to 0.45] | (0.03)  [0.27 to 0.41] |
|  |  |  |  |
| Covariates | ✓ | ✓ | ✓ |
|  |  |  |  |
| Observations | 5000 | 2451 | 2540 |
| R² | 0.20 | 0.21 | 0.17 |

Unstandardized beta-coefficients are reported, robust standard errors in parentheses, 95% CI in square brackets; *** p<0.001, ** p<0.01, * p<0.05, + p<0.10; Covariates include sex (if applicable), age, state, employment status, marital status, education, self-rated health and number of chronic conditions.

Supplementary Table 4. Determinants of loneliness (total sample and stratified by sex). Results of multiple linear regressions.

|  | (1) | (2) | (3) |
| --- | --- | --- | --- |
| Independent variables | Loneliness – Total sample | Loneliness - Men | Loneliness - Women |
|  |  |  |  |
| Dimension psychosocial impact: difficulty doing your usual job | 0.46*** | 0.47*** | 0.45*** |
|  | (0.03)  [0.40 to 0.52] | (0.04)  [0.39 to 0.55] | (0.05)  [0.36 to 0.54] |
|  |  |  |  |
| Covariates | ✓ | ✓ | ✓ |
|  |  |  |  |
| Observations | 5000 | 2451 | 2540 |
| R² | 0.18 | 0.21 | 0.16 |

Unstandardized beta-coefficients are reported, robust standard errors in parentheses, 95% CI in square brackets; *** p<0.001, ** p<0.01, * p<0.05, + p<0.10; Covariates include sex (if applicable), age, state, employment status, marital status, education, self-rated health and number of chronic conditions.

Supplementary Table 5. Determinants of loneliness (total sample and stratified by sex). Results of multiple linear regressions (all covariates are displayed)

|  | (1) | (2) | (3) |
| --- | --- | --- | --- |
| Independent variables | Loneliness – Total sample | Loneliness – Men | Loneliness - Women |
|  |  |  |  |
| Oral health-related quality of life | 0.12*** | 0.12*** | 0.12*** |
|  | (0.00) | (0.00) | (0.00) |
| Sex: - Female (Reference category: Male) | -0.04 |  |  |
|  | (0.52) |  |  |
| - Diverse | -0.46 |  |  |
|  | (0.37) |  |  |
| Age | -0.02*** | -0.02*** | -0.02*** |
|  | (0.00) | (0.00) | (0.00) |
| Federal state: - Bavaria (Baden-Württemberg) | -0.05 | 0.02 | -0.13 |
|  | (0.60) | (0.88) | (0.36) |
| - Berlin | -0.15 | -0.30 | 0.12 |
|  | (0.34) | (0.13) | (0.61) |
| - Brandenburg | -0.19 | -0.04 | -0.32 |
|  | (0.26) | (0.86) | (0.17) |
| - Bremen | -0.03 | -0.02 | -0.07 |
|  | (0.89) | (0.96) | (0.84) |
| - Hamburg | -0.09 | -0.43 | 0.24 |
|  | (0.66) | (0.12) | (0.39) |
| - Hesse | -0.24** | -0.40** | -0.05 |
|  | (0.05) | (0.02) | (0.79) |
| - Mecklenburg-Western Pomerania | -0.21 | -0.46* | 0.05 |
|  | (0.27) | (0.07) | (0.86) |
| - Lower Saxony | -0.01 | 0.01 | -0.02 |
|  | (0.94) | (0.92) | (0.89) |
| - North Rhine-Westphalia | -0.13 | -0.15 | -0.10 |
|  | (0.18) | (0.26) | (0.46) |
| - Rhineland-Palatinate | 0.27* | 0.31* | 0.24 |
|  | (0.05) | (0.08) | (0.25) |
| - Saarland | -0.21 | -0.48 | 0.05 |
|  | (0.42) | (0.24) | (0.87) |
| - Saxony | -0.26* | -0.14 | -0.35* |
|  | (0.07) | (0.48) | (0.09) |
| - Saxony-Anhalt | -0.13 | -0.12 | -0.16 |
|  | (0.45) | (0.60) | (0.55) |
| - Schleswig-Holstein | -0.41** | -0.37 | -0.45* |
|  | (0.01) | (0.12) | (0.05) |
| - Thuringia | -0.37** | -0.44* | -0.36 |
|  | (0.03) | (0.06) | (0.14) |
| Marital status: Living together: married/partnership (Reference category: Other including single, divorced, widowed, living separated: married/partnership) | -0.56*** | -0.69*** | -0.45*** |
|  | (0.00) | (0.00) | (0.00) |
| Education: - medium education (Ref.: low education) | -0.07 | -0.03 | -0.12 |
|  | (0.43) | (0.80) | (0.38) |
| - high education | -0.30*** | -0.15 | -0.46*** |
|  | (0.00) | (0.28) | (0.00) |
| Employment status: Retired (Reference category: Full-time employment) | -0.15* | -0.06 | -0.28** |
|  | (0.09) | (0.62) | (0.04) |
| - Other | 0.07 | 0.07 | 0.03 |
|  | (0.28) | (0.50) | (0.74) |
| Self-rated health (from 1 = very poor to 5 = very good) | -0.65*** | -0.77*** | -0.55*** |
|  | (0.00) | (0.00) | (0.00) |
| Number of chronic diseases | 0.07*** | -0.01 | 0.15*** |
|  | (0.00) | (0.71) | (0.00) |
| Constant | 6.53*** | 7.03*** | 6.08*** |
|  | (0.00) | (0.00) | (0.00) |
|  |  |  |  |
| Observations | 5,000 | 2,451 | 2,540 |
| R² | 0.20 | 0.23 | 0.18 |

Unstandardized beta-coefficients are reported, p-values in parentheses; *** p<0.001, ** p<0.01, * p<0.05, + p<0.10

Supplementary Table 6. Determinants of loneliness (total sample and stratified by sex). Results of multiple linear regressions (with quartiles for oral health-related quality of life; all covariates are displayed)

|  | (1) | (2) | (3) |
| --- | --- | --- | --- |
| Independent variables | Loneliness – Total sample | Loneliness – Men | Loneliness - Women |
|  |  |  |  |
| Oral health-related quality of life: - Second quartile (Ref.: Lowest quartile) | 0.35*** | 0.25** | 0.44*** |
|  | (0.00) | (0.05) | (0.00) |
| - Third quartile | 0.68*** | 0.66*** | 0.73*** |
|  | (0.00) | (0.00) | (0.00) |
| - Highest quartile | 1.27*** | 1.34*** | 1.22*** |
|  | (0.00) | (0.00) | (0.00) |
| Sex: - Female (Reference category: Male) | -0.06 |  |  |
|  | (0.28) |  |  |
| - Diverse | -0.36 |  |  |
|  | (0.50) |  |  |
| Age | -0.02*** | -0.02*** | -0.02*** |
|  | (0.00) | (0.00) | (0.00) |
| Federal state: - Bavaria (Baden-Württemberg) | -0.05 | 0.03 | -0.13 |
|  | (0.64) | (0.81) | (0.36) |
| - Berlin | -0.11 | -0.26 | 0.13 |
|  | (0.45) | (0.19) | (0.59) |
| - Brandenburg | -0.18 | -0.01 | -0.34 |
|  | (0.27) | (0.97) | (0.15) |
| - Bremen | -0.00 | 0.04 | -0.05 |
|  | (0.99) | (0.92) | (0.87) |
| - Hamburg | -0.10 | -0.49* | 0.26 |
|  | (0.62) | (0.07) | (0.35) |
| - Hesse | -0.24* | -0.41** | -0.02 |
|  | (0.05) | (0.01) | (0.91) |
| - Mecklenburg-Western Pomerania | -0.26 | -0.50** | -0.00 |
|  | (0.17) | (0.04) | (0.99) |
| - Lower Saxony | -0.03 | -0.00 | -0.05 |
|  | (0.78) | (0.99) | (0.75) |
| - North Rhine-Westphalia | -0.13 | -0.14 | -0.11 |
|  | (0.18) | (0.30) | (0.43) |
| - Rhineland-Palatinate | 0.28** | 0.34* | 0.23 |
|  | (0.04) | (0.06) | (0.26) |
| - Saarland | -0.22 | -0.51 | 0.02 |
|  | (0.40) | (0.23) | (0.94) |
| - Saxony | -0.28* | -0.17 | -0.37* |
|  | (0.05) | (0.40) | (0.07) |
| - Saxony-Anhalt | -0.12 | -0.12 | -0.12 |
|  | (0.50) | (0.61) | (0.65) |
| - Schleswig-Holstein | -0.43** | -0.41* | -0.44* |
|  | (0.01) | (0.08) | (0.06) |
| - Thuringia | -0.38** | -0.45** | -0.38 |
|  | (0.02) | (0.05) | (0.10) |
| Marital status: Living together: married/partnership (Reference category: Other including single, divorced, widowed, living separated: married/partnership) | -0.55*** | -0.69*** | -0.42*** |
|  | (0.00) | (0.00) | (0.00) |
| Education: - medium education (Ref.: low education) | -0.09 | -0.02 | -0.16 |
|  | (0.33) | (0.90) | (0.23) |
| - high education | -0.29*** | -0.12 | -0.45*** |
|  | (0.00) | (0.37) | (0.00) |
| Employment status: Retired (Reference category: Full-time employment) | -0.15 | -0.04 | -0.29** |
|  | (0.10) | (0.76) | (0.03) |
| - Other | 0.06 | 0.06 | 0.01 |
|  | (0.36) | (0.56) | (0.88) |
| Self-rated health (from 1 = very poor to 5 = very good) | -0.60*** | -0.71*** | -0.51*** |
|  | (0.00) | (0.00) | (0.00) |
| Number of chronic diseases | 0.08*** | -0.00 | 0.15*** |
|  | (0.00) | (0.94) | (0.00) |
| Constant | 6.19*** | 6.67*** | 5.74*** |
|  | (0.00) | (0.00) | (0.00) |
|  |  |  |  |
| Observations | 5,000 | 2,451 | 2,540 |
| R² | 0.20 | 0.23 | 0.19 |

Unstandardized beta-coefficients are reported, p-values in parentheses; *** p<0.001, ** p<0.01, * p<0.05, + p<0.10

Supplementary Table 7. Determinants of loneliness (total sample). Results of multiple linear regressions (with interaction term: sex x oral health-related quality of life; all covariates are displayed)

|  | (1) |
| --- | --- |
| Independent variables | Loneliness – Total sample |
|  |  |
| Oral health-related quality of life | 0.12*** |
|  | (0.00) |
| Sex: - Female (Reference category: Male) | -0.07 |
|  | (0.33) |
| Interaction term: Oral health-related quality of life x female (Ref.: male) | 0.01 |
|  | (0.36) |
| Age | -0.02*** |
|  | (0.00) |
| Federal state: - Bavaria (Baden-Württemberg) | -0.05 |
|  | (0.60) |
| - Berlin | -0.14 |
|  | (0.35) |
| - Brandenburg | -0.19 |
|  | (0.26) |
| - Bremen | -0.05 |
|  | (0.84) |
| - Hamburg | -0.07 |
|  | (0.73) |
| - Hesse | -0.24** |
|  | (0.05) |
| - Mecklenburg-Western Pomerania | -0.21 |
|  | (0.27) |
| - Lower Saxony | -0.01 |
|  | (0.94) |
| - North Rhine-Westphalia | -0.13 |
|  | (0.18) |
| - Rhineland-Palatinate | 0.27* |
|  | (0.05) |
| - Saarland | -0.21 |
|  | (0.43) |
| - Saxony | -0.26* |
|  | (0.07) |
| - Saxony-Anhalt | -0.14 |
|  | (0.43) |
| - Schleswig-Holstein | -0.41** |
|  | (0.01) |
| - Thuringia | -0.38** |
|  | (0.02) |
| Marital status: Living together: married/partnership (Reference category: Other including single, divorced, widowed, living separated: married/partnership) | -0.56*** |
|  | (0.00) |
| Education: - medium education (Ref.: low education) | -0.07 |
|  | (0.43) |
| - high education | -0.30*** |
|  | (0.00) |
| Employment status: Retired (Reference category: Full-time employment) | -0.15* |
|  | (0.09) |
| - Other | 0.07 |
|  | (0.29) |
| Self-rated health (from 1 = very poor to 5 = very good) | -0.65*** |
|  | (0.00) |
| Number of chronic diseases | 0.07*** |
|  | (0.00) |
| Constant | 6.54*** |
|  | (0.00) |
|  |  |
| Observations | 4,991 |
| R² | 0.20 |

Unstandardized beta-coefficients are reported, p-values in parentheses; *** p<0.001, ** p<0.01, * p<0.05, + p<0.10
